# Real-time imaging of nanoscale electrochemical Ni etching under thermal conditions†

**DOI:** 10.1039/d0sc06057g

**Published:** 2021-02-26

**Authors:** Shu Fen Tan, Kate Reidy, Julian Klein, Ainsley Pinkowitz, Baoming Wang, Frances M. Ross

**Affiliations:** Department of Materials Science and Engineering, Massachusetts Institute of Technology MA 02139 Cambridge USA fmross@mit.edu

## Abstract

The ability to vary the temperature of an electrochemical cell provides opportunities to control reaction rates and pathways and to drive processes that are inaccessible at ambient temperature. Here, we explore the effect of temperature on electrochemical etching of Ni–Pt bimetallic nanoparticles. To observe the process at nanoscale resolution we use liquid cell transmission electron microscopy with a modified liquid cell that enables simultaneous heating and biasing. By controlling the cell temperature, we demonstrate that the reaction rate and dissolution potential of the electrochemical Ni etching process can be changed. The *in situ* measurements suggest that the destabilization of the native nickel oxide layer is the slow step prior to subsequent fast Ni removal in the electrochemical Ni dissolution process. These experiments highlight the importance of *in situ* structural characterization under electrochemical and thermal conditions as a strategy to provide deeper insights into nanomaterial transformations as a function of temperature and potential.

## Abstract 2

The combination of electrochemical analysis, temperature control and *in situ* TEM imaging directly probes the etching of Ni from bimetallic Ni–Pt nanoparticles.

## Graphical abstract

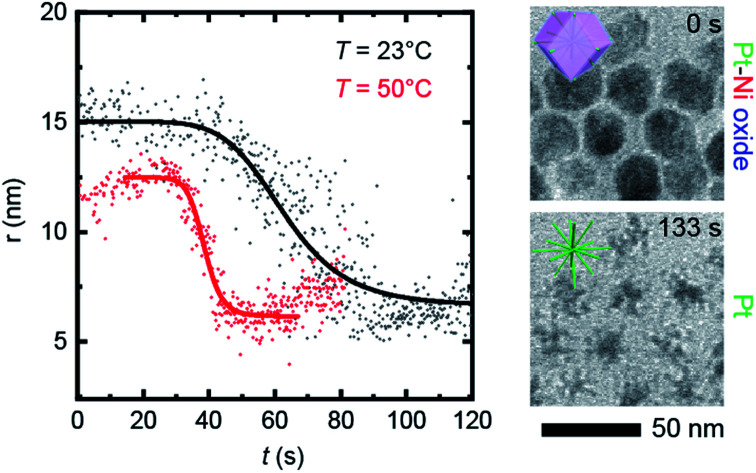

## Introduction

Temperature is known to provide a powerful control knob in tuning the reaction pathways and final products of electrochemical reactions. Small differences in temperature can cause dramatic changes in reaction mechanisms^1,2^ and make new reaction pathways accessible, such as intercalation processes at elevated temperature.^3^ Temperature has a particularly important role in battery reactions: for example, lithium ion batteries show slow kinetics at low temperatures and materials degradation at higher temperatures, both of these affecting performance, safety, and cycle life.^4^ Multiple effects can play a role simultaneously: for corrosion processes, elevated temperatures increase the tendency for material oxidation and sulfidation while also speeding up the diffusion rate and mass transport rate between the material's surface and its environment.^5^ As a result, the outcome of many electrochemical reactions is temperature-dependent. This includes, for example, the morphology and composition of nanostructures formed by galvanic reactions.^6^ Control of temperature during electrochemical reactions is also useful in quantitative analysis. Measurements over a range of temperature can provide thermodynamic and kinetic parameters such as standard reduction potentials, activity coefficients and equilibrium constants,^7^ an approach that has proved useful in technological applications ranging from sensing^8^ to biotechnology.^9^ At high temperatures, the mass transport rate *via* diffusion or convection is greatly enhanced and often increases current signals compared to measurements at ambient temperature.^10–12^ Thus, the use of temperature as a variable parameter in electrochemical analysis provides benefits such as lowering detection limits^13,14^ and increasing the sensitivity of systems with sluggish kinetics.^15,16^

We are particularly interested in using temperature as a variable to increase the opportunities for structural and compositional control of electrochemically-produced nanostructures. Temperature control should be especially useful in processes where both chemical and electrochemical reactions play a role. This is the case when creating catalytically active nanoparticles (NPs). These nanostructures are formed by first synthesizing bimetallic NPs then selectively removing one of the component elements to change the surface composition and morphology, thereby tuning the catalytic properties. As an example, the surface area and catalytic activity of Ni–Pt NPs can be increased by removing the non-noble metal (Ni) to form a Pt-rich surface. Both chemical and electrochemical etching can change the surface composition and have been used separately.^17^ Chemical processing is often difficult to control due to random site etching,^18,19^ but electrochemical processing is a relatively straightforward procedure to remove the Ni progressively.^20,21^ Varying the reaction temperature could in principle change the balance between chemical and electrochemical processes and hence allow the tuning of surface composition and overall morphology. To establish whether temperature can indeed be a useful control variable for processing bimetallic NPs, we need to understand the interplay of these etching phenomena under different conditions. In particular, how do the etching kinetics of the NPs change at elevated temperature, and what structures form under each condition? A quantitative view of temperature effects is critically needed for optimizing the nanostructures produced.

To address this challenge we use a technique, liquid cell transmission electron microscopy (TEM), that enables processes in liquid media to be imaged at high spatial and temporal resolution.^22–24^ We adapt the liquid cell for simultaneous biasing and heating of the sample by incorporating electrodes and a heater circuit. Electrodes have been used in liquid cells since the initial development of liquid cell TEM, resulting in detailed measurements of the structural evolution of nanomaterials under electrochemical bias.^25–30^ Indeed, liquid cell TEM has already been applied to visualize the etching of mono-^31^ and bimetallic NPs.^32–34^ For Ni–Pt NPs in particular, liquid cell microscopy has revealed an oxide-controlled etch mechanism under chemical and electrochemical control;^35^ for other NPs, multiple, complex dissolution pathways are observed dependent on surface facet orientation,^31^ strain^34^ and shell structure.^32,33^ Separating the chemical and electrochemical effects to achieve quantitative understanding and control of etching has proved challenging. Meanwhile, the incorporation of heater circuits into liquid cells has enabled studies of processes at elevated temperatures such as core–shell structure formation^36^ and thermally-induced shrinkage of microgels in water.^37^ The combination of heating and electrical biasing is expected to provide a unique probe of the dynamics of electrochemical processes under a broader range of conditions than has been possible so far, evaluating the complex processes at work.

We first describe the design of an electrochemical heating liquid cell then use its capabilities to image the electrochemical etching of Ni–Pt NPs. Compared to prior studies of these Ni–Pt NPs, we highlight the role of temperature as a control knob for adjusting the electrochemical parameters of the etching reaction. We model the performance of the heating electrochemical liquid cell to establish the temperature and potential across the field of view, identifying regions where the electrode process predominantly takes place during heating. The ability to localize reactions within the cell is an advantage in distinguishing between reaction pathways. By combining imaging, electrochemical measurement and modelling, we show that cell temperature plays a central role in tuning the kinetics and potential of the nanoscale electrochemical etch processes and we discuss the pathways that dominate the reaction. Temperature acts as a key variable to control both the rate of the etching process and the onset potential, suggesting promising future opportunities for nanostructure control.

## Results and discussion

The starting structures were Ni–Pt NPs chemically synthesized^38^ in the shape of rhombic dodecahedra with well-defined facets. These nanostructures have been shown to exhibit high efficiency as oxygen reduction reaction catalysts.^39^ The procedure for synthesizing NPs capped with oleylamine and loading them into a liquid cell holder is described in Experimental section. Etching was carried out in 0.1 M aqueous sulfuric acid (H_2_SO_4_) electrolyte solution at various temperatures under potentiostatic and cyclic voltammetry conditions.

**Fig. 1:**
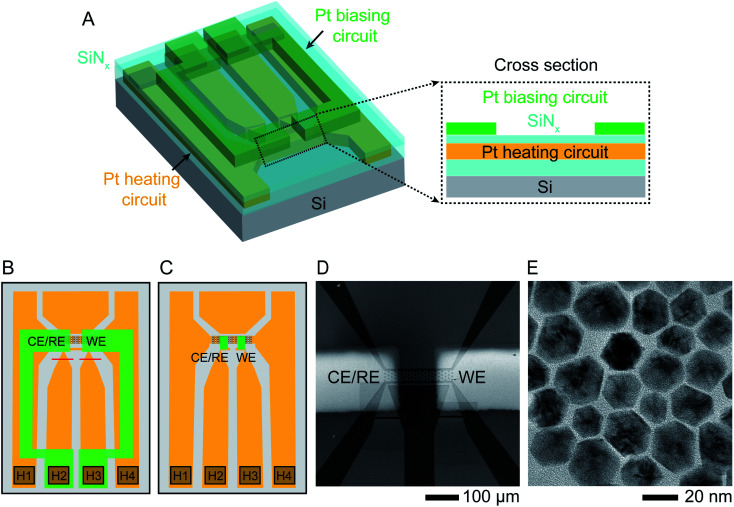
Experimental set-up. Schematic showing the modified liquid cell chip. (A) 3D cross section and (B) schematic top view of commercially available heater chip^40^ with 50 nm thick Pt resistance heater crossing the viewing window (squared region in B) and connected to two contact pads H1, H4 in the lower corners. The heater circuit (indicated in orange) is encapsulated by SiN_*x*_ above and below (cyan). Two 30 nm thick Pt electrodes (green) are electron-beam deposited using a shadow mask to overlap the viewing window. The electrodes are contacted at pads H2 and H3 and isolated from the heater circuit by the upper SiN_*x*_ layer. Red lines show two FIB cuts that separate the two central contact pads since these were originally connected. (C) A similar design using smaller Pt electrodes (green) with dimensions 50 μm × 20 μm, connected to the external contacts with windows etched through the upper SiN_*x*_ layer. (D) Scanning Electron Microscope (SEM) image of the viewing window of design (B) displaying the two Pt electrodes overlapping the heater. (E) TEM image of oleylamine-capped Ni–Pt rhombic dodecahedron (RD) nanoparticles (NPs).

The liquid cell was designed to enable simultaneous heating and electrical biasing during the removal of Ni from the NP surface. The chips were fabricated starting from standard commercial heating chips,^40^ which heat the liquid by flowing current through a Pt resistance heater that passes across the electron-transparent silicon nitride viewing window. The heater is fully encapsulated by SiN_*x*_ layers above and below (Fig. 1A). The chips were modified by electron beam deposition through a shadow mask to pattern two 30 nm thick Pt electrodes over the two edges of the viewing window. Two different geometries used in our experiments are shown in Fig. 1B and C. Fig. 1D displays an SEM image of the viewing window and electrodes. This simple design is capable of simultaneous heating and electrochemical biasing using a two-electrode system comprised of a working electrode and combined reference/counter electrode. A quasi-reference electrode, used in room temperature electrochemical liquid cells, is not included here due to the limited number of electrical connections. Heating and electrochemical parameters are controlled separately: a sourcemeter at constant current controls the heater circuit and a potentiostat controls the electrodes, as described in Experimental section. After testing for electrical isolation between the heater and electrode circuits, we drop-cast the NPs onto the chip, sealed the liquid cell then flowed electrolyte into the viewing window area. Initial characterization by scanning TEM (STEM) and energy-dispersive X-ray spectroscopy (EDX) chemical mapping show structure and composition consistent with what is expected from synthesis with atomic percentages of 90% Ni and 10% Pt (Fig. 1E and ESI Section 1†).

### Electrochemical characterization and room temperature reactions

In interpreting electrochemical liquid cell data it is important to first assess the stability of the system without bias, and with and without beam irradiation. The Ni–Pt NPs are stable in the electrolyte under the imaging conditions used, typically <30 e^−^ Å^−2^ s^−1^, without applied potential (ESI Section 2†). NiO is known to be unstable to etching at low pH, so the observed stability of Pt–Ni NPs appears surprising. However, it is consistent with previous observations,^35^ where Pt–Ni NP stability was explained through DFT calculations of the adsorption energy of O on Ni in the presence of Pt, leading to Pourbaix diagrams in which the O-covered surface is stable at lower pH in the presence of Pt.^35^

The experimental NP stability breaks down at higher electron flux, >150 e^−^ Å^−2^ s^−1^, and structural changes consistent with etching become visible over the experimental time frame (Fig. S2†). All data analyzed below was therefore obtained at low flux. The NPs are also stable if no electrolyte is present.

Initial characterization of the complete electrochemical system was carried out by measuring cyclic voltammograms. In a liquid cell filled with 0.1 M H_2_SO_4_ electrolyte, cyclic voltammograms measured in the presence of deposited NPs show the characteristics expected for the Pt electrodes as well as a small anodic peak at ∼200 mV observed in the first cycle (Fig. S3†). This is likely the Ni dissolution peak, based on the charge passed (∼10^−8^ C, consistent with the number of Ni atoms in the NPs at the electrode, as discussed in ESI Section 3†).

**Fig. 2:**
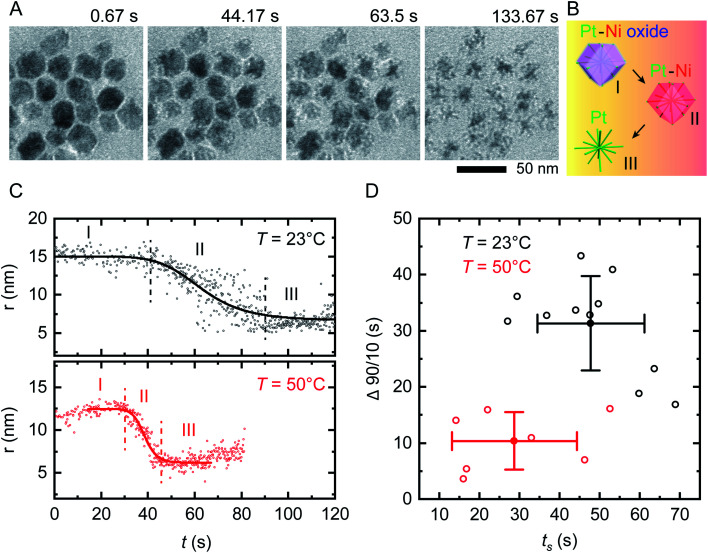
*In situ* electrochemical etching of Ni–Pt RD NPs in 0.1 M H_2_SO_4_ electrolyte solution upon applying constant potential of +500 mV at different temperatures. (A) Time series of *in situ* TEM images showing the dissolution of Ni during application of a 150 s long continuous positive potential of +500 mV at *T* = 23 °C inside a liquid cell with the electrode geometry shown in Fig. 1B (ESI Video 1†). (B) Schematic showing the etching stages of nanostructures: (I) destabilization of native nickel oxide layer, (II) electrochemical driven Ni dissolution and (III) remaining Pt skeleton. (C) Representative evolution of the NP radius as a function of time during electrochemical etching at *T* = 23 °C (black) and 50 °C (red), respectively. The solid lines represent sigmoid fits to the data. The vertically dashed lines define reaction start (*t*_s_) and end (*t*_e_). The small increase in measured radius in region I and III at *T* = 50 °C originates from slow drift in illumination conditions that change the image contrast. (D) Time evolution statistics from sigmoid fitting of 12 (8) individual NPs at *T* = 23 °C (*T* = 50 °C) showing the reaction duration (Δ90/10 = *t*_e_ − *t*_s_) as a function of *t*_s_. Corresponding mean values are plotted with standard deviations from statistical errors.

On applying a constant potential we expect Ni to be oxidized first due to its negative standard reduction potential of −260 mV.^41^ Previous studies of similar Ni–Pt bimetallic NPs have shown that application of +500 mV is sufficient to destabilize the passivated Ni oxide layer and lead to electrochemical dissolution of Ni.^35^ This voltage is also consistent with values obtained from electrochemical studies of Ni corrosion in nanocrystalline thin films immersed in sulfuric acid.^42^Fig. 2A shows the result of applying a positive bias of +500 mV at room temperature using our modified liquid cell. After a delay of 10–20 s, Ni starts to disappear from the NP surface in a relatively isotropic manner, transforming RD NPs at *t* = 44 s into tetradecapod NPs at *t* = 133 s. The schematic in Fig. 2B illustrates the electrochemical Ni dissolution process. To confirm that the observed etching is driven by the applied bias, note that NPs at the working electrode are etched while NPs at the counter/reference electrode are unchanged during the experiment (ESI Section 4†). We attribute the time delay to the time needed to destabilize the Ni oxide passivated layer. After Ni removal, the NPs appeared stable because the reduction potential of Pt^2+^/Pt (1.18 V *vs.* SHE) is higher and positive compared to Ni.

We can summarize this electrochemical Ni etching process in three stages. (I) Destabilization of the passivated native Ni oxide film on the NP surface (eqn (1)). We expect that the hydrogen involved in this reaction is generated electrochemically through electrolysis of the dilute H_2_SO_4_ electrolyte (see ESI Section 3† for CVs). (II) Active dissolution of Ni on NPs at the working electrode (eqn (2)) with a series of reduction reactions at the counter electrode (eqn (3)–(5)), eventually followed by (III) Formation of Ni sulfate^43^ from ions in the bulk solution, leaving behind the Pt skeleton (eqn (6)). The overall etching process takes place even in areas not irradiated by the beam, implying that radiolytic generation of species such as H_2_ are not necessary.

1 Stage I: NiO + H_2_ → Ni + 4H_2_O

Stage II:

2 WE: Ni → Ni^2+^ + 2e^−^

3 CE: 4H_2_SO_4_ + 2e^−^ → SO_4_^2−^ + SO_2_ + 2HSO_4_^−^ + 2H_3_O^+^

4 O_2_ + 2H_2_O + 4e^−^ → 4OH^−^

5 2H^+^ + 2e^−^ → H_2_

6 Stage III: Ni^2+^ + SO_4_^2−^ → NiSO_4_

### Reactions at elevated temperature

We first assess the stability of the system under beam irradiation and heating with no bias. ESI Section 5† shows that the NPs in electrolyte are stable under irradiation during heating, without applied potential. Even up to *T* = 90 °C, minimal changes are seen in the NPs after multiple hours. (On heating without liquid in the cell, the NPs are stable up to *T* = 300 °C.) We now conduct the same electrochemical etching experiment as in Fig. 2A but at elevated temperature. Fig. 2C shows the etching of two NPs at *T* = 23 °C and *T* = 50 °C. The parameter shown is the radius that corresponds to the projected area measured for each NP at each time, as determined by image processing based on computer vision techniques (Experimental section). This measure ignores irregularities and leads to some systematic error but does not alter the observed trend, as discussed in Fig. S6.† Note that the electrochemical Ni etching on Ni–Pt NPs takes place in an isotropic manner as shown in Fig. 2A, thus we measure the NP radius to obtain the best estimate of Ni removal. Tracking details are described in Fig. S6D and E.† From the radius *vs.* time plots for each NP at each temperature, we determine the etching start (*t*_s_) and end times (*t*_e_) by fitting a sigmoid function, with *t*_s_ and *t*_e_ defined by the 90/10 rule. From both times we determine the overall reaction duration, Δ90/10 = *t*_e_ – *t*_s_. Fig. 2D shows that at *T* = 23 °C, *t*_s_ = 47.8 s with standard deviation 13.4 s and Δ90/10 = 31.3 ± 8.4 s, while at *T* = 50 °C, *t*_s_ = 28.7 ± 15.6 s and Δ90/10 = 10.4 ± 5.1 s. Further details are provided in the ESI: Fig. S7† shows *in situ* TEM images of tracked NPs, Fig. S8† shows the remaining 11 data sets at 23 °C and Fig. S9† shows the remaining 7 data sets at 50 °C. Overall, there is scatter in the etching of individual NPs but the onset *t*_s_ and duration Δ90/10 of the electrochemical etching reaction at *T* = 50 °C are about half as long as at *T* = 23 °C. Electrochemical Ni dissolution takes place even more quickly at *T* = 70 °C, concluding in <6 s (*i.e.* Δ90/10 < 6 s) but bubble formation hindered our imaging and quantification process (ESI Section 7†).

We further explore the electrochemical parameters of the etching process *via* benchtop experiments shown in Fig. S12.† The measurement was similar to that in Fig. 2, applying a constant potential of +500 mV for 150 s at different temperatures, but using the smaller electrodes illustrated in Fig. 1C. The total charge passed in each experiment (*Q*), which corresponds to the total material processed by the electrochemical reaction, increases with temperature. We would expect *Q* to be independent of temperature, since it represents etching of all the Ni present; furthermore, the total charge passed during the etching time is much higher than expected given the expected volume of NPs etched (ESI Section 8†). This therefore suggests other chemical reactions or leakage across the SiN_*x*_ window.^44^ A reasonable possibility is oxidation of the capping agent oleylamine, since it has been reported that oleylamine is unstable during electrochemical cycling at potentials higher than 1.0 V per RHE.^45^ These results emphasize the benefits of *operando* imaging methods: electrochemical analysis alone does not readily distinguish between Ni dissolution and side reactions such as oxidation of capping agents, whereas images are most sensitive to the removal of metallic species, with changes in the surfactant not highly visible. The combination of electrochemical analysis and *in situ* imaging provides a more complete picture of the etching process.

### Etching potential

**Fig. 3:**
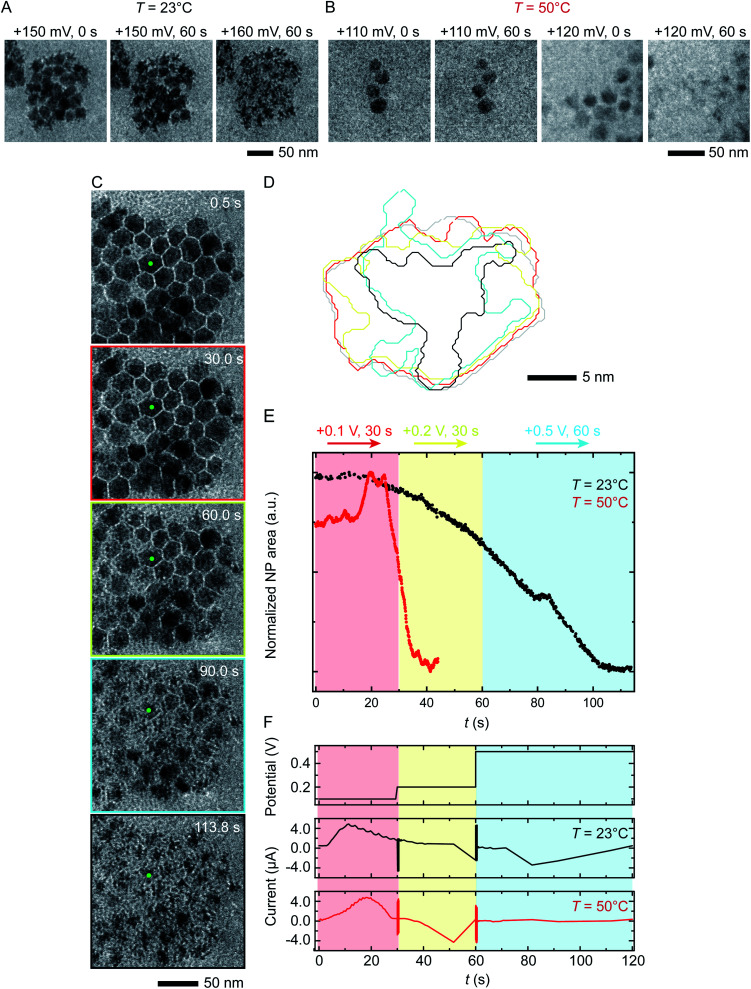
Electrochemical etching of Ni–Pt RD NPs in 0.1 M H_2_SO_4_ during chronoamperometry at different temperatures. Time series of *in situ* TEM images showing electrochemical Ni dissolution in a liquid cell filled with 0.1 M H_2_SO_4_ electrolyte on application of continuous bias of (A) +150 and 160 mV for 60 s at *T* = 23 °C and (B) +110 and 120 mV for 60 s at *T* = 50 °C. (C) Images extracted from a TEM movie (ESI Video 2†) showing Ni dissolution at *T* = 23 °C. The electrode configuration in Fig. 1B was used and the red, yellow and cyan outlines correspond to the potential, respectively 100 mV for 30 s, 200 mV for 30 s and 500 mV for 60 s. (D) Corresponding perimeter contour plot for the NP indicated in green. (E) Normalized area *vs.* time for NPs during reaction at *T* = 23 °C (black line) and *T* = 50 °C (red line). (F) Potential and current at *T* = 23 °C and 50 °C under the same chronoamperometry conditions but recorded using the electrode configuration in Fig. 1C.

We next discuss another key factor expected to depend on temperature: the potential required for etching. To test the effect of temperature on etching potential, we first examine the onset potentials for *T* = 23 °C and 50 °C by gradually increasing the potential in 10 mV, 60 s steps over the range +100 mV to +200 mV. Time series of TEM images in Fig. 3A and B clearly show the onset of electrochemical Ni dissolution. The onset varies with temperature, occurring at +160 mV and +120 mV for *T* = 23 °C and 50 °C, respectively, suggesting that a series of steps can be designed to control the reaction kinetics.

Temperature-dependent etch potentials are also evident in double-step chronoamperometry experiments. We pre-treat the electrochemical cell by holding the potential at +100 mV for 30 s (a point at which the cell shows zero faradaic current), then step the potential to +200 mV for 30 s then +500 mV for 60 s, recording movies, contour plots and areas of individual NPs. At both *T* = 23 °C and 50 °C (Fig. 3C–E), Ni dissolution does not occur at the +100 mV pre-potential. However, at both temperatures a cathodic reduction peak is visible (Fig. 3F). We attribute this to the reduction of the Ni oxide surface layer (Fig. 2, stage I). The presence of the cathodic reduction peak is consistent with our proposed mechanism in which destabilization of the native nickel oxide layer^35^ is the slow step that precedes Ni removal during electrochemical dissolution of Ni–Pt NPs.

In the subsequent potential steps, Ni dissolution is visible. At *T* = 23 °C the reaction is slow at +200 mV (Fig. 3C and E; also Fig. S10† for binary images). This is expected because the potential is only a short way above the onset potential of +160 mV. At +500 mV dissolution takes place more quickly. The total current decreases over the course of the experiment (Fig. 3F, black line). At *T* = 50 °C, a rapid decrease of NP area is visible at +200 mV (Fig. 3E, red line). The +200 mV potential at *T* = 50 °C is further above the onset at +120 mV, consistent with a faster reaction than at the same potential at *T* = 23 °C. Very little further change was visible during the period at +500 mV, suggesting that the electrochemical reaction is complete after the +200 mV period, unlike the case at *T* = 23 °C. These observations show that (1) the etching reaction rate can be varied by controlling the etch potential, and (2) the etch potential can be tuned by varying temperature. These results are consistent with literature^46^ showing that the potential required for electrochemical etching is lower at elevated temperature. This is further supported by an electrochemical characterization using ferricyanide/ferrocyanide redox couple at different *T*_s_ (ESI Section 9†) where clearly the potential is shifted to lower values as a function of *T*.

Although we expect the standard potential required for any electrochemical reaction to change with temperature,^47^ over the temperature range accessed here these particular reactions (eqn (2)) are not expected to change by more than 10 mV.^46^ However, the equations used for calculating the temperature dependence include approximations: they are for water at 298.15 K and are accurate only for zero standard isobaric heat capacity change.^46^ In the non-standard conditions of the liquid cell experiments, these approximations may not hold.^46^ The relatively large change we observe in onset potential suggests that the temperature effect may in fact be underestimated by the calculations. In practical terms, the effect of temperature on the etching onset potential and reaction rate suggests that temperature can be used as a control knob for the dissolution reaction.

### Liquid cell design for reaction rate control

We have shown that electrochemical Ni dissolution of Ni–Pt NPs occurs more rapidly at higher temperatures and at larger applied positive voltages. A feature of liquid cell design is that over the region visible in the electron microscope, variations in the experimental conditions are present. In particular, we find that the NP etching phenomenon in Fig. 2 and 3 is visible for NPs located on the working electrode and within ∼10 μm from the edge of this electrode. NPs located >20 μm away from the working electrode remain unetched (Fig. S14†).

**Fig. 4:**
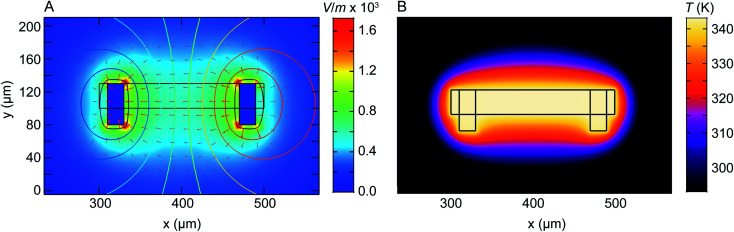
Numerical modelling of the electric field distribution and heat map within the heating electrochemical cell. The electrode configuration is as in Fig. 1C and the heater is assumed to coincide with the window (central rectangle). (A) Plot of electric field distribution (V m^−1^) as a colour map when 100 mV is applied to the working electrode (right) with respect to the counter/reference electrode (left). Contours placed every 10 mV represent electric potential while arrow length is proportional to current density. (B) Plot of temperature distribution between the two Pt electrodes. Although electrically isolated from the heater, the electrodes modify the temperature distribution because of their higher thermal conductivity compared to the SiN_*x*_ and Si that surround the window.

In the case of liquid cell experiments involving both heating and biasing, spatial variations may have an even stronger effect on the experiment. To interpret the electrochemical data from the experiment it is important to evaluate variations in both temperature and potential. As a first step to quantify local conditions we carried out a finite-element method calculation of the distribution of heat, electric potential, and electric field simultaneously. A COMSOL Multiphysics stationary two-dimensional finite element simulation^48^ was used to extract information along the Pt electrodes and in the electrolyte. Boundary conditions for window temperature and electrode potentials are given in Experimental section. The simulation solved partial differential equations for both the electromagnetic and heat transfer phenomena. Fig. 4 shows the simulated electric field (false colour map), electric potential (contour), and current density (arrows) for the cell with smaller electrodes in Fig. 1C. The simulation shows a localized enhancement of the current density and a non-uniform electric field distribution along the Pt working electrode surface. In particular, a local potential enhancement is visible at the corners of the electrode, extending ∼10 μm into the surrounding electrolyte. The difference in electric field (potential) for regions 10 μm and 20 μm away from the electrode can be up to 580 V m^−1^ (50 mV) as displayed in Fig. 4A. The larger electrode configuration in Fig. 1B shows a similar trend except that the field is more concentrated between the electrodes (Fig. S15†). The heat distribution is comparatively homogeneous throughout the window and electrode areas (Fig. 4B).

Overall, the temperature and electric fields are independent – for example, heat transfer does not depend on the potential, as compared for 200 mV and 500 mV in Fig. S16.† The combined potential and temperature fields are expected to enhance the rate of electrochemical reactions in specific locations, consistent with our observations. This may be considered as challenging if we are trying to directly correlate an image series obtained at one location with electrochemical parameters such as the total current or charge passed throughout the entire cell. However, we suggest that this selectivity can be an advantage if the experiment is designed appropriately. Most liquid cell designs include regions of the electrode that extend out of the window area. Reactions can occur at these locations but cannot be imaged. If heating takes place only at the window, as in the chip design used here, then it is possible to confine the entire electrochemical reaction to the visible area of the chip, with the consequent benefits in relating total current flow or charge passed to the materials process that is visible *in situ*.

## Experimental section

### Sample preparation

Oleylamine (cat. no. O7805-100G, Sigma-Aldrich Co., St Louis, MO, USA), hydrogen hexachloroplatinate(iv) hexahydrate (H_2_PtCl_6_·6H_2_O) (cat. no. 206083-1G, Sigma-Aldrich Co., St Louis, MO, USA), nickel(ii) nitrate hexahydrate (Ni(NO_3_)_2_·6H_2_O) (cat. no. 203874-20G, Sigma-Aldrich Co., St Louis, MO, USA), sulfuric acid (cat. no. 1090721000-1L, EMD Millipore Co., Billerica, MA, USA) and hexane (cat. no. 296090-1 L, Sigma-Aldrich Co., St Louis, MO, USA) were used as received without further purification. All aqueous solutions were prepared using deionized water.

We synthesized the Pt–Ni nanoparticles (NPs) using a protocol adapted from Niu *et al.*^38^ An aqueous solutions of 400 μL of 0.1 g mL^−1^ H_2_PtCl_6_·6H_2_O and 350 μL of 0.1 g mL^−1^ Ni(NO_3_)_2_·6H_2_O were mixed with 1 mL of oleylamine. A transparent green precursor solution was obtained after water removal by heating at 120 °C under stirring for 1 hour. 10 mL of oleylamine was pre-heated in a two-necked flask at 160 °C under nitrogen purging for 1 hour. The temperature of oleylamine was raised and once it reached 245 °C, the green precursor solution was injected. After stirring for about 1 hour, the solution turns from green to yellow, brown and finally, black. Aliquots were taken 3–5 minutes after the solution turned black. All aliquots were washed at least five times by centrifugation at 12 000 rpm for 5 minutes and re-suspending in a 1 : 1 (v/v) of hexane : ethanol mixture by sonication. The washed particles were re-dispersed in hexane.

### Experimental procedure

For *in situ* electrochemical cell experiments, ∼500 nL of the Pt–Ni NP solution was loaded into the liquid cell. Each cell consisted of one 100 nm-spacer chip with 30 nm-thick SiN_*x*_ window (Hummingbird Scientific, Lacey, WA, USA) and a modified heating chip with microfabricated electrodes directly on top of the 50 nm-thick SiN_*x*_ window (Hummingbird Scientific, Lacey, WA, USA). Four connections were made to (1) the Pt working electrode, (2) the Pt counter/reference, and (3 & 4) the Pt heating circuit. Before assembling the cell and loading the solution between the windows, we cleaned the chips with an oxygen plasma for ∼1 minute to render the SiN_*x*_ surfaces hydrophilic. Each cell was placed in an analytical flow holder (Hummingbird Scientific, Lacey, WA, USA). After checking for leaks, the holder was inserted into a JEOL 2011 TEM operated at 200 kV for *in situ* imaging. We introduced the sulfuric acid aqueous solution (0.1 M H_2_SO_4_) into the liquid cell *via* a flow tube (diameter of 200 μm and length of 50 cm) connected to a syringe pump at a flow rate of 5 μL min^−1^. It takes a few minutes for the solution to reach the window area of the electrochemical cell. During imaging the incident electron flux ranged from 10 to 30 e^−^ (Å^2^ s)^−1^. Image series were acquired at 6–10 frames per second using an AMT camera (Advanced Microscopy Techniques Corp., Woburn, MA, USA). The electrochemical measurements were recorded using a Gamry Reference 600+ potentiostat (Gamry Instruments, Pennsylvania, USA) while heating was performed using the software and controller provided by Hummingbird Scientific with temperature calculated based on the resistivity of the heating element. *t* = 0 in the videos is the time that we applied the bias voltage to the Pt–Ni NPs. Post-growth, high-angle annular dark-field scanning TEM (HAADF-STEM) imaging and chemical analysis were performed with a JEOL 2010F operated at 200 kV.

### Image processing

The images in Fig. 2 and 3 were obtained by analysing image sequences from TEM movies in Python 3.7 (ref. 49) using the libraries numpy,^50^ opencv^51^ and matplotlib.^52^ Each image (frame) is imported as 8 bit grayscale (0 to 255) matrix. Data are smoothed and salt-and-pepper noise is reduced by using a bilateral filter with a diameter of 9. Individual NPs are determined in the filtered images using a binary threshold and contours are obtained using the provided function for finding contours in the opencv^51^ library. The areas of individual NPs are obtained either directly from the number of pixels within each contour or by fitting a minimum enclosing circle.

### Finite-element method simulation

We used COMSOL Multiphysics^48^ software to perform finite element analysis on the heating-biasing liquid cell chip. A 2D stationary model was created using the electric currents (ec) and heat transfer in solids (ht) modules. For the smaller electrode design in Fig. 1C, the geometry was defined by a 200 μm × 30 μm rectangular region as the window, as well as two 20 μm × 50 μm regions on either side of the window as the electrodes. In the case of the larger electrode system in Fig. 1B, the electrodes were defined as 200 μm × 350 μm rectangular regions. Using data for dielectric permittivity of H_2_SO_4_ and water, an average *ε* was used to describe the 0.1 M H_2_SO_4_ electrolyte.^53^ The electrodes were set to material properties of solid Pt. A physics controlled mesh was set with a ‘normal’ element size, which made use of an automatic mesh refinement at the boundaries. Assuming quasi–static equilibrium, the initial voltage of the right electrode was set at 0.1 V, 0.2 V or 0.5 V, with the left electrode grounded with respect to the rest of the chip. The outer edges of the chip were set to be electrically insulated and the remainder of the interior boundaries were set to the continuity condition. These were enforced *via* boundary conditions. Voltage contours were set every 0.01 V. Solving the electrostatics equations *via* the finite element method resulted in the electric fields, potential maps, and current density in Fig. 4A. In the case of the heat transfer equations in Fig. 4B, all boundaries around the edge of the chip were set to room temperature and out of plane heat flux was added to simulate the heat sink of the surrounding Si. The 200 μm × 30 μm window area was set to 70 °C. The remainder of the boundaries were set to continuity conditions, with the heat capacity *C*_p_, thermal conductivity *k*, and density *ρ* obtained from literature for Pt^54^ and water^55^ used to model the electrodes and surrounding area, respectively. The COMSOL calculations, in many cases, were cross-checked to ensure accuracy using the MATLAB FEA Physics Simulation Toolbox^56^ with the same boundary conditions.

## Conclusions

We carried out *in situ* electrochemical Ni etching on Ni–Pt NPs as a model electrocatalyst system under conditions of elevated temperature and were able to measure the reaction kinetics at different cell temperatures based on both imaging in the electrochemical heating cell and the simultaneous electrochemical response. Our observations reveal that (1) it is possible to tune the electrochemical Ni dissolution rate by changing the cell temperature; (2) by controlling the cell temperature, the potential at which etching is initiated can be shifted to lower magnitude than is required at ambient temperature. Simulations of the liquid cell geometry showed that (3) the electrochemical etching process is site-dependent, making it possible to localize reactions within the cell. The observations and simulations together reveal how the cell temperature affects the electrochemical etching kinetics and etch potential with a benefit of distinguishing between electrochemical etching and side reactions. This type of information is difficult to obtain from *ex situ* studies that lack the combination of temporal and spatial resolution. The experimental capability for control of potential and temperature provides the opportunity to explore etching at a range of temperature and potential in order to improve our control of this useful material processing step. We anticipate future opportunities for *in situ* systems where the flow of the electrolyte, the cell temperature, and the potential can be controlled in a similar manner to that used in the conventional electrochemical reactors used to control the structure of bimetallic electrocatalysts.

The ability to localize reactions at places where potential and temperature reach the conditions required for a reaction to take place may enable more precise electrochemical interpretation, for example to differentiate between a desired reaction and side reactions by comparing image contrast changes with the overall response (current or charge passed) in the liquid cell. An important component of such future electrochemical heating cells will be to go beyond the two-electrode configuration to include a reference electrode, necessitating additional electrical connections but improving the quantification of electrochemical data. More generally, *operando* electrochemical imaging is already useful in accessing a range of processes such as battery reactions and corrosion. We anticipate that modulation of the electrochemical cell temperature will provide opportunities to explore these and other electrochemical reactions using the unique time-resolved imaging capabilities of liquid cell TEM.

## Author contributions

S. F. T. conceived the project and designed and performed experiments and data analysis. K. R. performed simulations. J. K. wrote and executed codes for data analysis. A. P. contributed to chip design and provided electrochemical interpretation. B. W. performed sample processing. F. M. R. supervised the project. All authors discussed the results and co-wrote the manuscript.

## Conflicts of interest

There are no conflicts to declare.

## Supplementary Material

SC-012-D0SC06057G-s001

SC-012-D0SC06057G-s002

SC-012-D0SC06057G-s003
