# Supplementary material for: Real-time imaging of nanoscale electrochemical Ni etching under thermal conditions
Source: Chem Sci. 2021 Feb 26;12(14):5259–68. doi: 10.1039/d0sc06057g (PMC8179569; doi:10.1039/d0sc06057g)
Supplement: SC-012-D0SC06057G-s001 [file SC-012-D0SC06057G-s001.pdf]

Electronic Supplementary Material (ESI) for Chemical Science.
This journal is © The Royal Society of Chemistry 2021

Supporting Information for

Real-Time Imaging of Nanoscale Electrochemical Ni Etching under Thermal Conditions

Shu Fen Tan, Kate Reidy, Julian Klein, Ainsley Pinkowitz, Baoming Wang and Frances M. Ross*

Department of Materials Science and Engineering, Massachusetts Institute of Technology, MA 02139, Cambridge, USA.

*Correspondence: fmross@mit.edu

Table of Contents

1. Energy-dispersive X-ray spectroscopy (EDX) chemical maps 2
2. Beam effects 2
3. Cyclic voltammograms 3
4. Spatial variation in reaction rates in the liquid cell 4
5. Thermal stability of Pt-Ni NPs 4
6. Image analysis 4
7. Bubble formation during electrochemical etching at 70 °C 7
8. Chronoamperometry 8
9. Cyclic voltammetry of ferricyanide/ferrocyanide redox couple 8
10. Variation in potential and temperature across the liquid cell 9
11. Supporting videos captions 11
12. Supporting references 11

1. Energy-dispersive X-ray spectroscopy (EDX) chemical maps

The image and EDX chemical maps in Figure S1 suggest that the NPs contain a Pt skeleton covered by the non-noble metal Ni as well as a surface layer of Pt surrounding the entire NP, with atomic percentages of 90% Ni and 10% Pt.

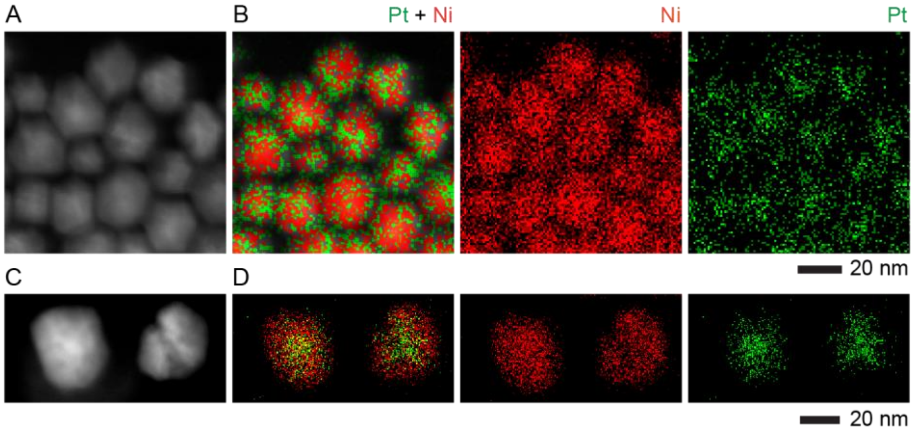

Figure S1: (**A**, **C**) STEM images and (**B**, **D**) the corresponding EDX maps of Pt-Ni RD NPs.

2. Beam effects

Liquid cell TEM imaging, Figure S2, indicates that NPs show beam-induced changes at a high electron flux of 150 $e^-/Å^2·s$ but not at a lower flux of 10-30 $e^-/Å^2·s$.

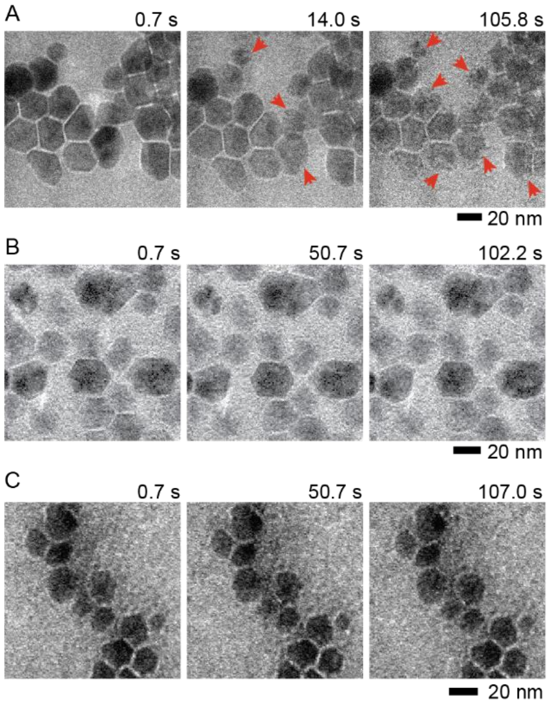

Figure S2: Time series of *in situ* TEM images showing beam-induced changes (arrows) in Pt-Ni RD NPs at (**A**) 150 $e^-/Å^2·s$, but no visible changes at (**B**) 30 $e^-/Å^2·s$ or (**C**) 10 $e^-/Å^2·s$, upon flowing 0.1 M H_2SO_4 electrolyte solution at 23 °C.

3. Cyclic voltammograms

The cyclic voltammogram (CV) in Figure S3A (recorded with deposited Pt-Ni NPs in 0.1 M H_2SO_4 electrolyte solution at T = 23 °C, using a three-electrode liquid cell chip where ITO/Pt serves as WE and two Pt electrodes serve as CE and RE) shows the characteristic voltammetric profile of the Pt electrode.[1] We assign Peaks I and II to hydrogen desorption and absorption and Peaks III and IV to Pt oxide reduction and Pt oxidation peaks respectively. We attribute the positive slope in the baseline to the high solution resistance of the thin (~300 nm) electrolyte layer that results in a large IR drop.[2]

This can be compared to a CV (Figure S3B) collected using the two-electrode configuration. The anodic peak (Peak I) at ~0.2 V is likely the oxidation peak of Ni because it disappeared after the first CV cycle, presumably because most or all of the Ni in the NPs was dissolved during the first cycle. Furthermore, the integrated area under Peak I is ~3 x 10^{-8} C, which is consistent with our expectation for the charge flow during electrochemical Ni dissolution: we estimate 10^{-8} C for dissolving 10^{10} Ni atoms, calculated using a particle diameter and density of 400 NP/μm^2 (from TEM images) over the electrode area plus a 10-μm wide strip around the electrode (a total of 40 μm x 50 μm).

To assign the other features in Figure S3B, we expect the electrolysis of the dilute sulfuric acid to first proceed through oxidation of SO_4^{2-} ions to SO_2 molecules and O^{2-} ions, after which O^{2-} ions further oxidize to H_2O at the CE. The increase at positive potential (Peak II) is likely due to the oxygen evolution reaction arising from dilute sulfuric acid electrolysis. During the subsequent CV cycle shown in red, Peak III appears and Peak II is suppressed. Although we cannot assign Peak III unambiguously, we suggest that it arises from hydrogen evolution reactions that are expected under acidic conditions,[3, 4] and are limited by the amount of hydrogen species in the liquid cell volume. We believe this electrochemically generated hydrogen is the main source of hydrogen in Eq. 1 during the etching reaction of the NPs. Note that another source of H_2 molecules is radiolysis. Gas bubbles were observed at elevated *T*s if potential or current is applied for longer periods, presumably because the hydrogen created electrochemically and by radiolysis exceeds its solubility in water.[5] However, since NP etching occurs even without the beam, radiolysis cannot be the major source of hydrogen.

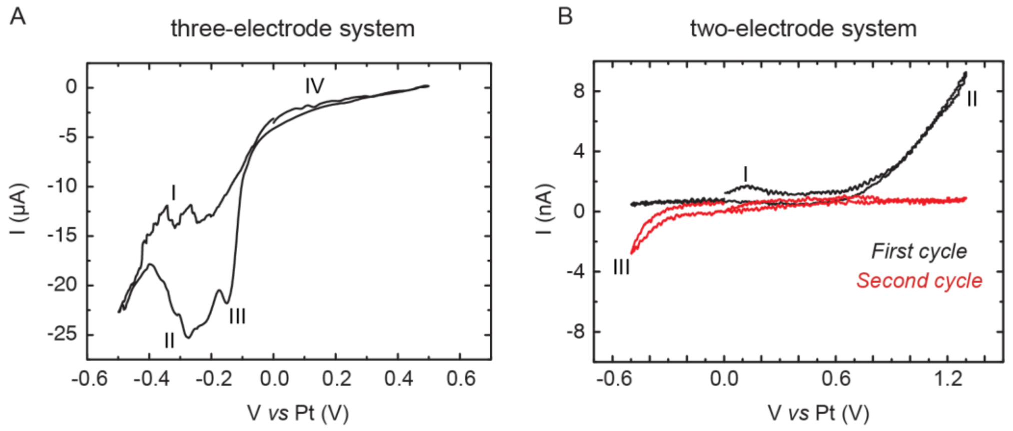

Figure S3: Cyclic voltammograms acquired in 0.1 M H_2SO_4 in the presence of Pt-Ni NPs in (**A**) a three-electrode (ITO/Pt WE, Pt CE and RE) electrochemical liquid cell with a large WE area (2000 μm^2) and scan rate of 10 mV s^{-1} and (**B**) the two-electrode system in Figure 1C with a scan rate of 20 mV s^{-1} . The difference in the magnitude of the current is assumed to arise from the different electrode areas and materials.

4. Spatial variation in reaction rates in the liquid cell

To verify that etching is driven by bias, we compared morphological changes in Pt-Ni NPs near the WE and the CE/RE. After applying a positive bias of 500 mV, the NPs at the CE/RE remained essentially unchanged (Figure S4A) while the NPs at the WE transformed into Pt skeletons (Figure S4B). These results confirm that Ni removal is driven by the electrochemical reaction.

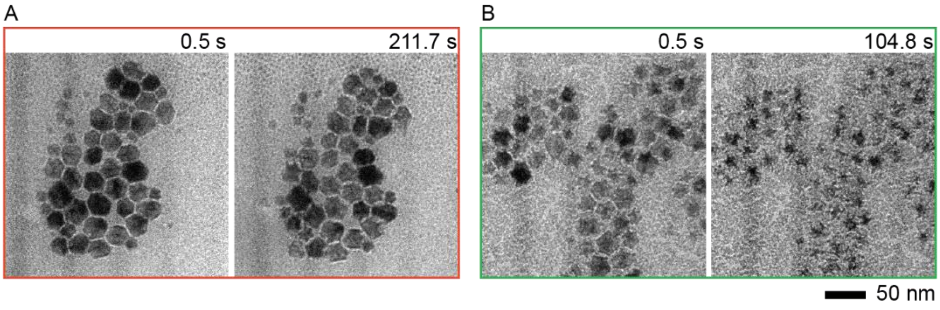

Figure S4: Time series of *in situ* TEM images showing the Pt-Ni NPs before and after applying positive bias of +500 mV for the times shown near (**A**) the CE/RE (red) and (**B**) the WE (green) in 0.1 M H_2SO_4 electrolyte.

5. Thermal stability of Pt-Ni NPs

We checked the thermal stability of the NPs at different temperatures and zero bias while flowing the 0.1 M H_2SO_4 electrolyte solution. It has been shown that these NPs are stable in 0.1 M H_2SO_4 electrolyte solution due to the presence of Pt.[6] Figure S5A-C show that these Pt-Ni NPs are similarly stable at a range of cell temperatures.

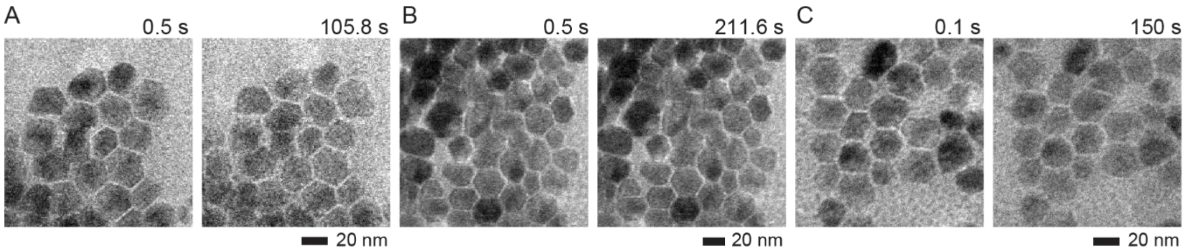

Figure S5: Time series of *in situ* TEM images showing the Pt-Ni NPs in an electrochemical cell with zero applied bias voltage while flowing 0.1 M H_2SO_4 electrolyte solution at cell temperature of (**A**) 23 °C, (**B**) 50 °C and (**C**) 70 °C.

6. Image analysis

Results of the image processing described in Experimental Section are shown in Figures S6-10. Figure S6A shows a typical NP with the contour derived from image processing (blue) and the corresponding fit of the contour with a minimum enclosing circle (red). We define the minimum enclosing circle as the smallest circle that surrounds the profile. The areas derived, $A_{contour}$ and A_{fit}, are illustrated schematically in Figure S6B. Figure S6C shows the agreement between areas for a NP tracked over time. We conclude that the minimum enclosing circle and contour tracking both provide similar measures of the reaction progress. Figure S6D-E shows

the typical evolution of a NP with both the minimum enclosing circle and contour tracking during electrochemical etching and the corresponding schematics. We fit the contour with a minimum enclosing circle to measure the NP radius in order to obtain the best estimate for Ni removal after adjusting the image threshold so as to exclude the Pt “arms” in these measurements. The non-zero value of radius at the end of the analysis is due to the remaining dense Pt in the center. Subtracting this base value would be possible but would not affect fits or extracted parameters.

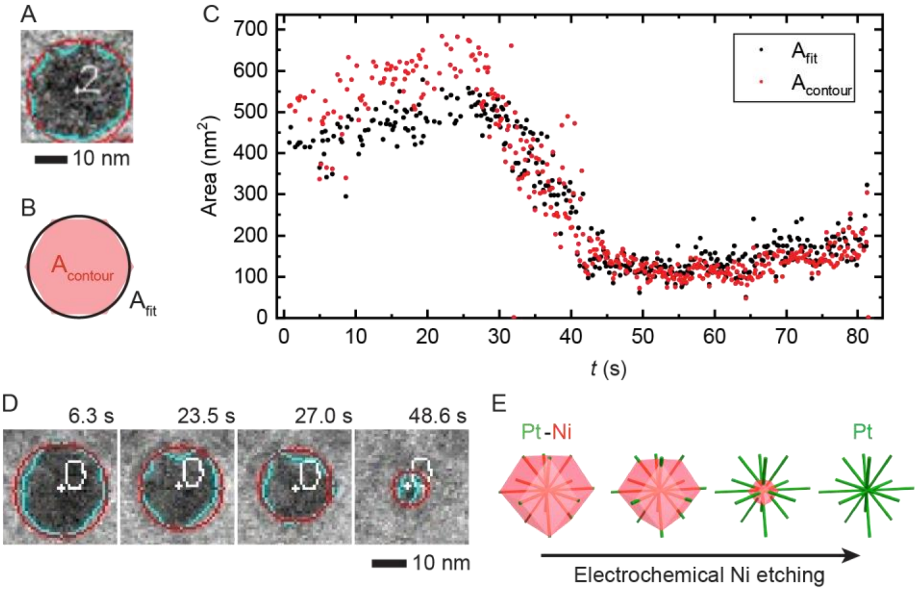

Figure S6: (**A**) TEM image and (**B**) schematic showing area fitted and area tracked by contour. (**C**) Area *versus* time for areas fitted in our radius model (black) and areas tracked by contour (red). The rise at large *t* is due to slow changes in overall image brightness. (**D**) Time series of *in situ* TEM images show area fitted and area tracked by contour. (**E**) Schematic illustrating the structural and compositional changes of Ni-Pt NPs during electrochemical etching.

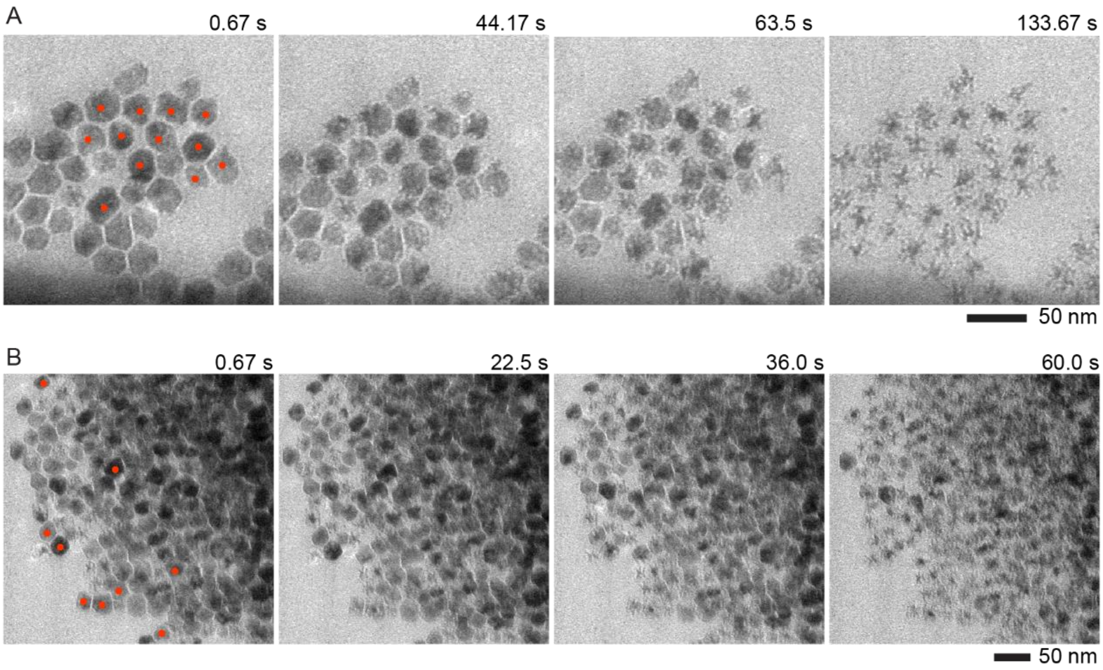

Figure S7: Time series of *in situ* TEM images showing Ni dissolution during application of a 100-s long continuous positive potential of +0.5 V in a liquid cell filled with 0.1 M H_2SO_4 electrolyte solution at (**A**) T = 23 °C and (**B**) T = 50 °C. The red dots indicate the NPs that are tracked and shown in Figure 2D.

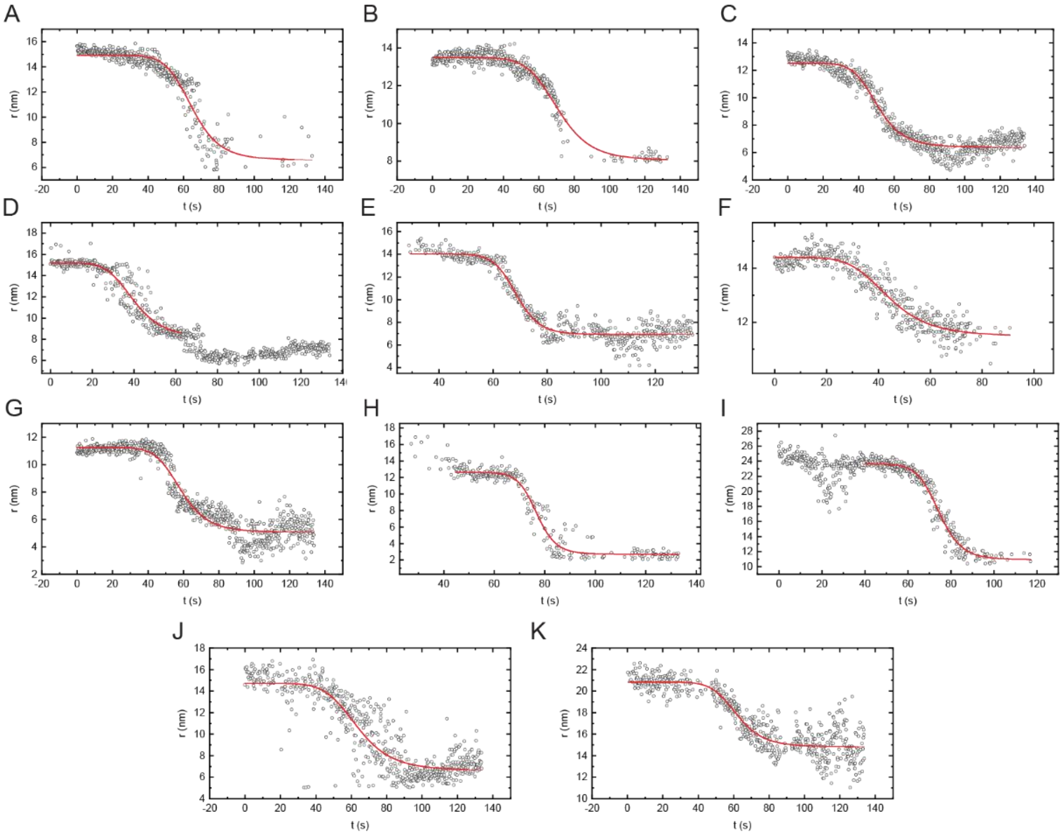

Figure S8: (**A-K**) Evolution of NP radius as a function of time during electrochemical etching at T = 23 °C. The red solid lines represent sigmoid fits to the data.

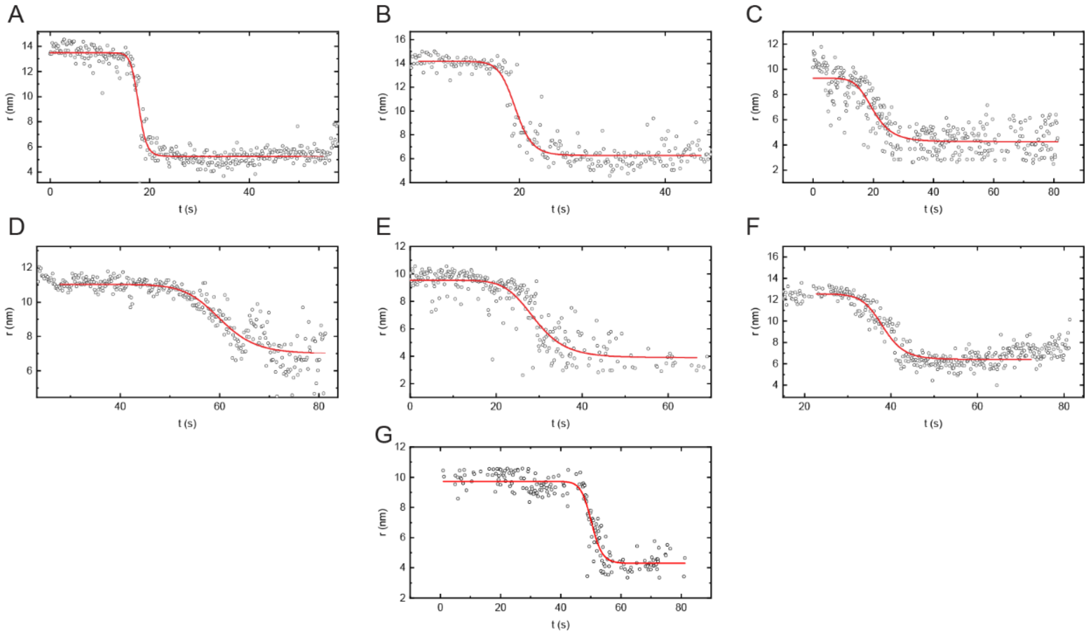

Figure S9: (**A-G**) Evolution of NP radius as a function of time during electrochemical etching at *T* = 50 °C. The red solid lines represent sigmoid fits to the data.

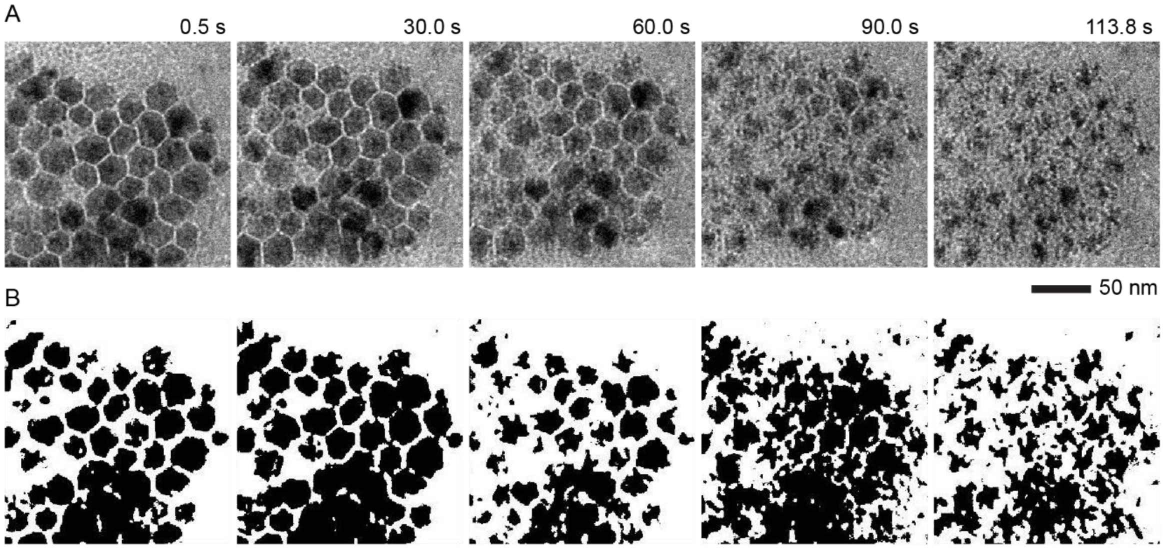

Figure S10: (**A**) Time series of *in situ* TEM images and (**B**) the corresponding binary images after thresholding showing the structural changes of Pt-Ni NPs as shown in main text Figure 3C.

7. Bubble formation during electrochemical etching at 70 °C

We consistently observed bubble formation at 70 °C as shown in Figure S11.

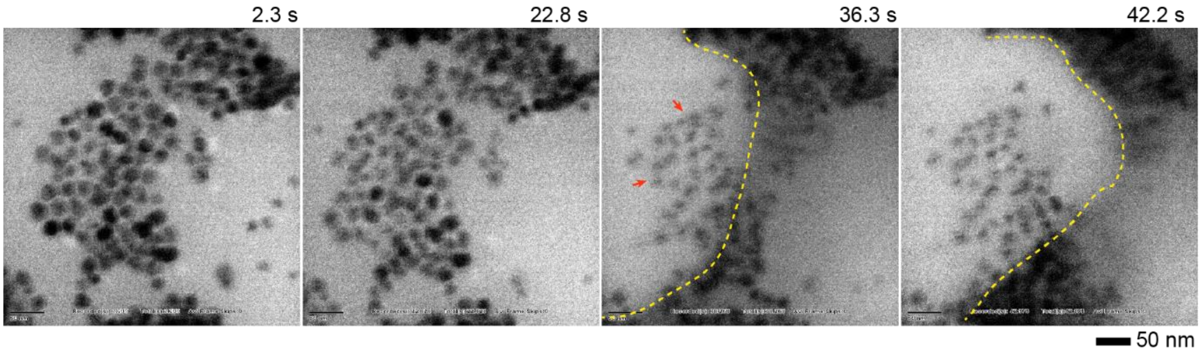

Figure S11: Time series of *in situ* TEM images showing electrochemical Ni dissolution in a liquid cell filled with 0.1 M H_2SO_4 electrolyte, on application of continuous positive bias of 0.5 V for 150 s at 70 °C. Red arrows indicate the beginning of dissolution whereas yellow dotted lines indicate the bubble position during imaging.

8. Chronoamperometry

Figure S12 shows the current response when +0.5 V is applied to the heating electrochemical liquid cell at different temperatures. The total charge passed is higher than expected from electrochemical etching of Ni (Section 3), suggesting that other chemical reactions or leakage current across the SiN_x window[7] may be contributing to the current recorded in chronoamperometry. The oxidation of oleylamine could be a source for the leakage current in Figure S12B as suggested by recent reports that oleylamine suffers from electrochemical cycling at potentials higher than 1.0 V/SHE.[8]

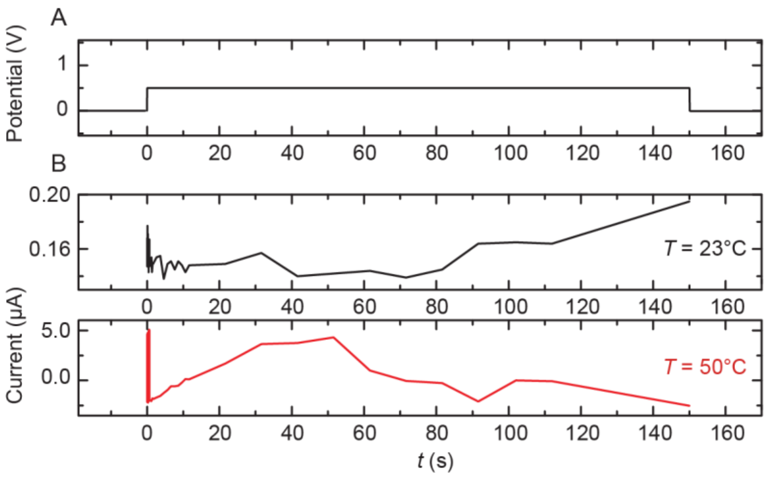

Figure S12: (**A**) Potential and (**B**) current response as a function of time during electrochemical etching at T = 23 °C (black) and T = 50 °C (red). Note the different current scales.

9. Cyclic voltammetry of ferricyanide/ferrocyanide redox couple

Figure S13A shows CVs acquired at 20 mV/s for $[Fe(CN)_6]^{3-/4-}$ (E^0 = +0.36 V vs SHE) using our two-electrode configuration at different temperatures. The CV collected at T = 50 °C has typical current peaks for a diffusion-controlled reaction. To satisfy the criteria of a Nernstian reversible electrochemical reaction, the peak potentials for anodic and cathodic (E_p^a

– E_p^c) should be equal to 59 mV/n where n is the number of electrons, and | i_p^a /i_p^c| should be unity. We measure (E_p^a – E_p^c) and | i_p^a /i_p^c| to be 50 mV (E_p^a = 0.05 V and E_p^c = 0 V with respect to Pt) and 29.4 which implies that the system deviates the from ideal reversible reaction.

However, the CVs collected at lower scan rates of 5 mV/s (Figure S13B) and 1 mV/s (Figure S13C) have a sigmoidal shape. This behaviour is expected for microelectrodes where the electrode dimension and diffusion distance are two competing factors on the time scale of the experiment.[9] There is enough material diffusing to the faces of the microelectrode to keep the current constant, resulting in levelling-out of the current and sigmoidal CVs. These results show good agreement with previous literature[10] suggesting that mass transport is dominated by diffusion at higher scan rate in microfluidic configurations with forced convection. At scan rate 1 mV/s, E_p^a and E_p^c are no longer measureable, thus we extract the half-wave potential ($E_{1/2}$) which is the midpoint from the CV curves at each T: 0.033 V at T = 23 °C, 0.011 V at T = 50 °C and 0.009 V at T = 70 °C. These results show that the potential is shifted to lower value as T increases, consistent with previously reported bench-top electrochemistry experiments.[11]

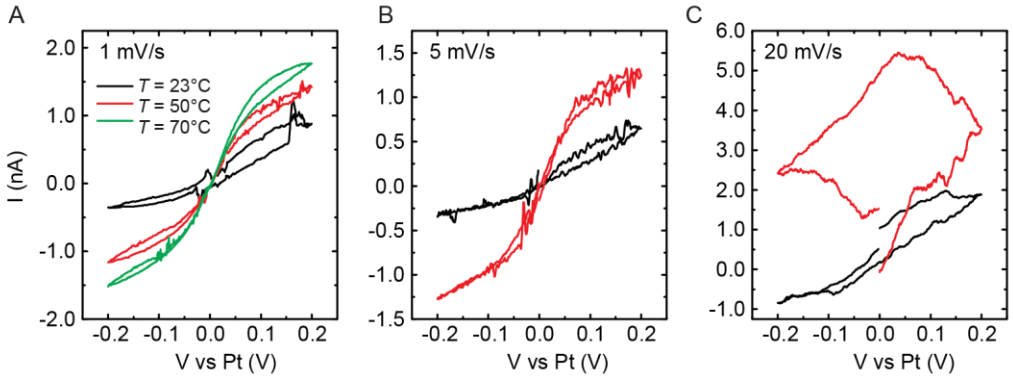

Figure S13: Cyclic voltammograms acquired in a electrochemical cell filled with 20 mM $K_4Fe(CN)_6$ + 0.1 M KCl at a flow rate of 10 µL/min using the two-electrode configuration in Figure 1C with a scan rate of (**A)** 20 mV s^{-1}, (**B**) 10 mV s^{-1} and (**C**) 1 mV s^{-1} at T = 23 °C, 50 °C and 70°C.

10. Variation in potential and temperature across the liquid cell

Figure S14 shows the reaction of Pt-Ni NPs on application of positive bias of 0.5 V for 150 s at 23 °C in 0.1M H_2SO_4 electrolyte solution, as a function of position with respect to the electrode. NPs located ~10 µm from the working electrode (Figure S14A-C) etch while NPs >20 µm from the working electrode (Figure S14D-F) remained unetched. This suggests that electrochemical reactions are possible up to 10 µm from the working electrode. The electric field and potential are calculated in Figures S15-16.

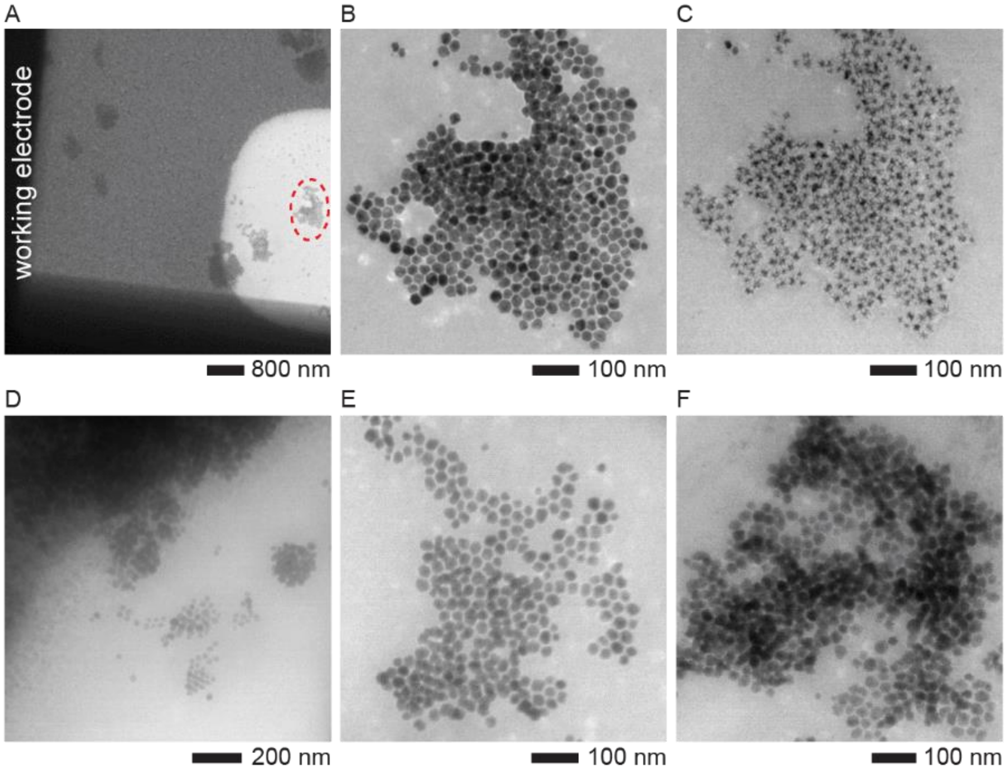

Figure S14: (**A**) Low magnification TEM image showing a cluster of Pt-Ni NPs (red dotted circle) that are ~10 µm away from the working electrode. TEM images showing those Pt-Ni NPs (**B**) before and (**C**) after applying positive bias of 0.5 V for 150 s in 0.1 M H_2SO_4 electrolyte. (**D-F**) Pt-Ni NPs further (> 20 µm) from the electrodes remain unchanged.

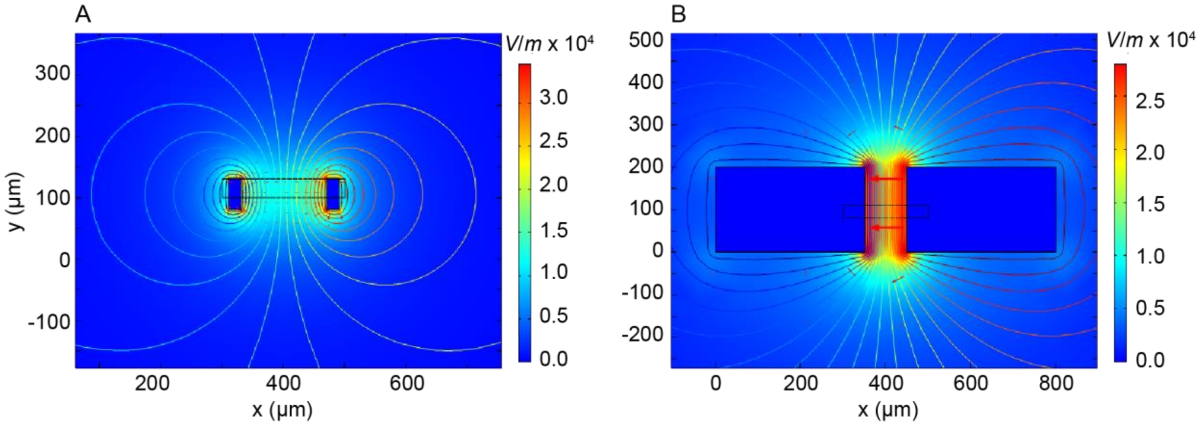

Figure S15: Numerical modelling of the electric field distribution for (**A**) small and (**B**) large electrodes. The field distribution (V/m) is shown as a false colour map where 0.1 V is applied to the WE with respect to the CE/RE. Contours shown every 0.01 V represent electric equipotentials (V), while arrow surface is proportional to current density.

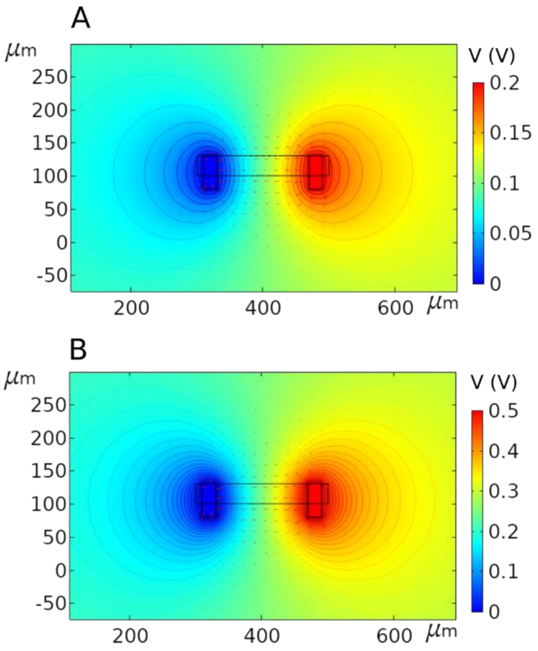

Figure S16: Simulated potential map when (**A**) 0.2 V and (**B**) 0.5 V is applied to the WE (right) with respect to the CE/RE (left) within the heating electrochemical cell. Equipotential contours are shown in increments of 0.01 V. Arrow surface represents relative current density.

11. Supporting videos captions

Video 1: Electrochemical etching of Ni-Pt nanoparticles in 0.1 M H_2SO_4 electrolyte solution upon applying constant potential of +500 mV at T = 23 °C.

Video 2: Electrochemical etching of Ni-Pt nanoparticles in 0.1 M H_2SO_4 during double-step chronoamperometry using +100 mV for 30 s, +200 mV for 30 s and +500 mV for 60 s at T = 23 °C.

12. Supporting references

1. G.-Z. Zhu, S. Prabhudev, J. Yang, C. M. Gabardo, G. A. Botton and L. Soleymani, *The Journal of Physical Chemistry C*, 2014, **118**, 22111-22119.
2. M. E. Holtz, Y. Yu, D. Gunceler, J. Gao, R. Sundararaman, K. A. Schwarz, T. A. Arias, H. D. Abruña and D. A. Muller, *Nano Lett.*, 2014, **14**, 1453-1459.
3. D. Zhan, J. Velmurugan and M. V. Mirkin, *J. Am. Chem. Soc.*, 2009, **131**, 14756-14760.
4. Y. Yokoyama, T. Fukutsuka, K. Miyazaki and T. Abe, *J. Electrochem. Soc.*, 2018, **165**, A3299-A3303.
5. J. H. Park, D. A. Steingart, S. Kodambaka and F. M. Ross, *Science Advances*, 2017, **3**, e1700234.
6. S. F. Tan, S. W. Chee, Z. Baraissov, H. Jin, T. L. Tan and U. Mirsaidov, *The Journal of Physical Chemistry Letters*, 2019, **10**, 6090-6096.
7. E. Fahrenkrug, D. H. Alsem, N. Salmon and S. Maldonado, *J. Electrochem. Soc.*, 2017, **164**, H358-H364.
8. I. A. Safo, C. Dosche and M. Özaslan, *Chemphyschem*, 2019, **20**, 3010-3023.
9. R. R. Unocic, R. L. Sacci, G. M. Brown, G. M. Veith, N. J. Dudney, K. L. More, F. S. Walden, D. S. Gardiner, J. Damiano and D. P. Nackashi, *Microsc. Microanal.*, 2014, **20**, 452-461.
10. A. J. Bard and L. R. Faulkner, *Electrochemical methods and applications*, Wiley-Interscience, New York; London, 2000.

11. C. Chu, B. W. Kwon, W. Lee and Y. Kwon, *Korean J. Chem. Eng.*, 2019, **36**, 1732-1739.
